# Are Narrow Focus Exhaustivity Inferences Bayesian Inferences?

**DOI:** 10.3389/fpsyg.2021.677223

**Published:** 2021-08-04

**Authors:** Alexander Schreiber, Edgar Onea

**Affiliations:** ^1^Department of Linguistics, SFB 1287, University of Potsdam, Potsdam, Germany; ^2^Institute of German Studies, University of Graz, Graz, Austria

**Keywords:** pragmatics, Bayesian models, rational speech act models, implicatures, focus, exhaustivity

## Abstract

In successful communication, the literal meaning of linguistic utterances is often enriched by pragmatic inferences. Part of the pragmatic reasoning underlying such inferences has been successfully modeled as Bayesian goal recognition in the Rational Speech Act (RSA) framework. In this paper, we try to model the interpretation of question-answer sequences with narrow focus in the answer in the RSA framework, thereby exploring the effects of domain size and prior probabilities on interpretation. Should narrow focus exhaustivity inferences be actually based on Bayesian inference involving prior probabilities of states, RSA models should predict a dependency of exhaustivity on these factors. We present experimental data that suggest that interlocutors do not act according to the predictions of the RSA model and that exhaustivity is in fact approximately constant across different domain sizes and priors. The results constitute a conceptual challenge for Bayesian accounts of the underlying pragmatic inferences.

## 1. Introduction

Interlocutors tend to interpret canonical sentences with narrow focus exhaustively when used as a direct answer to a congruent wh-question. For a dialogue as in (1-a) interlocutors, thus, draw the exhaustivity inference specified in (1-b).

(1)
  a. A: Who of the guests ate a cheeseburger?
    B: SKYLAR (ate a cheeseburger).
  b. Exhaustivity Inference: No other guest than Skylar ate a cheeseburger.

There are strong reasons to assume that exhaustivity inferences associated with narrow focus in such cases are not part of the conventional meaning of the respective assertions. Instead, they constitute implicatures or some sort of discourse inferences. We only give one argument here, but more can be found in the literature, cf. Krifka (2008), Zimmermann and Onea (2011), and Westera (2017) for more discussion and e.g., DeVeaugh-Geiss et al. (2015) for some relevant experimental results. In particular, it is by no means a contradiction to continue an answer such as in (1-a) by uttering a sentence containing an additive that would contradict the exhaustivity implicature as shown in (2-a). As opposed to this, the same continuation is blocked if an explicit exclusive like the English *only* is used in the first sentence which semantically encodes exhaustivity, as shown in (2-b).

(2)
  a. B: SKYLAR ate a cheeseburger. And Ashanti too.
  b. B': Only SKYLAR ate a cheeseburger. # And Ashanti too.

Even if not part of the semantic content, focus exhaustivity in question-answer patterns appears to be a strong default inference. As such, it can be and has been viewed on a par with scalar implicatures like (3). Some scholars suggested that scalar implicatures arise because of focus on the scalar item (e.g., Kuppevelt, 1996; Zondervan, 2010), others have suggested that focus inferences arise using the same grammatical mechanism as scalar implicatures in terms of exhaustifying alternative sets (e.g., Chierchia, 2013; Bade, 2016). Moreover, many scholars view both as some version of a quantity implicature (Geurts, 2010, cf. also Westera, 2017 for more discussion). This automatically raises the question how similar these inferences are in reality and how narrow focus exhaustivity inferences in question-answer constructions should be modeled.

(3) Bryce ate some of the cheeseburgers.
  Scalar Inference: Bryce did not eat all of the cheeseburgers.

Recent developments in pragmatic theory have strongly focused on devising predictive quantitative models of interlocutors behavior when confronted with implicatures. While there is a significant array of theoretical approaches on how such pragmatic reasoning should be modeled, one particular view has attracted much attention: the idea that pragmatic reasoning may be a prime example of social cognition. Following Baker et al. (2009), goal recognition associated with human action can be understood as rational Bayesian inverse planning. In line with this type of approach, Frank and Goodman (2012) provide a recursive model of goal recognition for referential games, further extended by Goodman and Stuhlmüller (2013) for scalar implicature^1^. We refer to this kind of framework as Rational Speech Act (RSA) framework. RSA models have been successful in modeling an ever growing number of pragmatic phenomena including scalar implicature (Goodman and Stuhlmüller, 2013), specificity implicatures and Horn implicatures (Bergen et al., 2012), and projection phenomena (Qing et al., 2016), generally achieving good fit between model predictions and experimental data. In the RSA framework, the interpretation of an expression *e* uttered by a speaker *S* toward an addressee *L* is the probability that *S* intends to refer to a state *s* given that he uttered *e*, denoted by *P*(*s*|*e*). This probability is obtained by Bayesian inference starting with the prior probability *P*(*s*) of the state *s* which is updated by the likelihood that *S* would have uttered *e* to refer to *s*, i.e., *P*(*e*|*s*). The latter depends mainly on what the literal meaning of *e* is, i.e., on what states are compatible with *e*, and what alternative statements could have been used to refer to various states compatible with the literal meaning of *e*. Thus, the interpretation depends on both the number and meaning of alternative expressions the addressee will consider and the number of possible states the speaker could in principle intend to refer to.

Interestingly, while scalar implicatures have been at the very core of research in the RSA framework, narrow focus exhaustivity has attracted much less attention. Here is a first hunch on why this would be the case: Although the RSA model appears to be entirely plausible from a logical perspective, from a cognitive perspective, there is a natural worry concerning the actual capacity of both speaker and addressee to consider the same set of alternatives and states in the computational process and to perform the computation correctly. For scenarios with very limited sets of alternatives, this may indeed be the case, but as the number of alternatives grows and computational complexity increases, mismatch ought to be more and more common. Still, there is no reason to assume that the reliability of the linguistic system in communication is severely alloyed by computational complexity.

In the phenomena that have been investigated so far with RSA models in the literature, the number of alternative states and expressions the speaker may employ was quite small. For example, the number of alternative states in the experiments of Bergen et al. (2012), Goodman and Stuhlmüller (2013), and Qing et al. (2016) ranges from two to four. The main difference between scalar implicatures and narrow focus exhaustivity is that the former always involves the same amount of alternatives whereas the number of relevant focus alternatives that need to be considered in the computation strongly differs. For (3) there are always exactly two alternative expressions that the interlocutors reason about, as shown in (4-a). But for (1-a), the number of alternatives depends on the set of contextually relevant individuals *D*_*C*_ as shown in 2. Moreover, the possible states interlocutors need to reason about also amount to a potentially huge number, as the number of possible combinations of guests that ate a cheeseburger increases exponentially with the number of guests. In the same vein, the prior probability of exhaustivity, i.e., of each individual partition cell defined by the question, decreases as well, as the number of contextually relevant alternatives increases. Hence, one could suspect that the potentially high number of alternatives relevant in the computation of focus exhaustivity may be an issue when applying the RSA framework for narrow focus exhaustivity.

(4)
  a. Alternatives for scalar implicatures: {“some”, “all”}
  b. Focus alternatives: {x ∈ *D*_*e*_|“x ate a cheeseburger”}; $D_{e}=P\left(D_{C}\right)$^2^

This leads us directly to the goal of this paper. We wish to investigate whether the RSA framework can be applied to narrow focus exhaustivity. This constitutes the main question of our paper. Whatever the answer to this question would be, we were also interested in the question what this tells us (a) about the RSA framework in general and (b) what this tells us about focus exhaustivity in general. These two questions are not unrelated because, as already mentioned above, exhaustivity in a number of phenomena has been argued to be based on focus, hence focus exhaustivity is indeed at the core of most exhaustivity phenomena in natural language. Therefore, it would be very surprising if people would behave “Bayesian” in other exhaustivity phenomena but not when interpreting narrow focus expressions.

The core of this paper is the presentation of two experimental studies we conducted in order to answer the main question. This is presented in section 3. The section is preceded by a brief introduction of our assumptions in terms of a theoretical background in section 2 and followed by an extended discussion in section 4 elaborating possible conclusions to be drawn from our experimental results.

Anticipating the results, our experimental data suggest that the RSA framework cannot be successfully applied to focus exhaustivity in the usual way, i.e., under the assumption that interlocutors reason about the connection between states in the world and rational choices of expressions via Bayesian inference. Instead, listeners seem to by and large ignore growing sets of alternatives and information on prior probabilities, leading to approximately constant exhaustivity.

If this is correct, one may suspect that completeness of answers to a question is a primitive notion of human social cognition that is radically divorced from the question how the world is in terms of states. While this conclusion seems compatible with the grammatical view of exhaustivity implicatures, as laid out in Chierchia et al. (2012), Chierchia (2013), and others, we refrain from pursuing a discussion of the way in which completeness of answers as postulated in this paper relates in detail to an implementation at the syntax-semantics interface. Rather, we see our results as a conceptual challenge to the modeling of linguistic phenomena like narrow focus exhaustivity inferences within Bayesian frameworks like the RSA framework.

## 2. Theory

In this section, we discuss the possibilities of the RSA-framework to deal with focus exhaustivity data. We, thereby, adopt the following strategy. After a brief introduction of the framework in some detail in section 2.1, we follow the most straightforward modeling recipe given the usual assumptions about the semantics of narrow focus in section 2.2. In particular, we assume, following Rooth (1992), Roberts (1996), Beaver and Clark (2008) and subsequent literature that in the context of a question the relevant focus alternatives for exhaustification are in fact the question alternatives in the sense of Hamblin (1973). Accordingly the possible states of the world that are needed to model exhaustivity are the exhaustified question alternatives, i.e., a partition in the sense of Groenendijk and Stokhof (1984). We build the model on the assumption that interlocutors in the RSA model reason about these alternatives. The predictions of the model are discussed in section 2.3. Since the predictions turn out to be somewhat contra-intuitive (though in some more abstract, mathematical sense entirely rational), we also wish to explore some alternative ways to model exhaustification of narrow focus in question-answer pairs within the RSA framework. In particular, in section 2.4 we will consider some possibilities which include either different model parameters or collapsing alternatives and states reflecting a simplification of cognitive burden on the interlocutors.

### 2.1. The Rational Speech Act Framework

As was done in other works using the RSA framework (Frank et al., 2009; Frank and Goodman, 2012, 2014; Goodman and Stuhlmüller, 2013; Goodman and Lassiter, 2015), we model human behavior connected with the interpretation of utterances by assuming three idealized pragmatic roles: A literal listener *L*_0_, who interprets all utterances literally, a Gricean speaker *S*_1_, who chooses his utterances based on the assumption that the listener is of type *L*_0_, and a Gricean listener *L*_2_, who assumes that the speaker is of type *S*_1_. The literal listener's belief *L*_0_(*s*|*e*) that the speaker refers to the state *s* by uttering the expression *e* is defined by

(5)
  $$\begin{array}{l} L_{0}\left(s|e\right)\propto ẽ\left(s\right)\cdot p\left(s\right) \end{array}$$

where *p*(*s*) is the prior probability of the state *s* and ẽ is defined by

(6)
  $$\begin{array}{l} ẽ\left(s\right)=\{\begin{array}{ll} 1, & \text{if }s\text{ is in the denotation of }e\\ 0, & \text{else} \end{array} \end{array}$$

The speaker *S*_1_ is assumed to act rationally according to Bayesian decision theory by maximizing the utility *U*(*e, s*) of his message *e* given a literal listener *L*_0_. Given that the speaker's goal is to find the best trade-off between being as informative as possible and using utterances that are as inexpensive as possible, utility can be defined by these two (usually antagonistic) factors in the following way:

(7)
  $$\begin{array}{l} U\left(e,s\right)=log\left(L_{0}\left(s|e\right)\right)-C\left(e\right) \end{array}$$

The first term represents the informativity of an utterance with respect to the speaker's intended state *s*. Equation (7) follows from the idea that informativity increases as the Kullback-Leibler divergence (Cover and Thomas, 2006) between the speakers beliefs concerning the distribution of states and the literal listener Equation (5) decreases. The second term *C*(*e*) represents the costs of uttering the message *e*.

The speakers choice can than be modeled by a *soft-max* function (Sutton and Barto, 1998):

(8)
  $$\begin{array}{l} S_{1}\left(e|s\right)\propto e^{\alpha \cdot U\left(e,s\right)} \end{array}$$

The decision noise parameter α ∈ [0, ∞] measures the speaker's deviation from optimal action selection. If α = ∞, the speaker always chooses the expression with highest utility, while α = 0 models a speaker who chooses among the true expressions completely randomly.

We assume further that the Gricean listener *L*_2_ can use Bayesian inference^3^ to recover the speaker's *S*_1_ intended state *s* given that the speaker uttered the expression *e*:

(9)
  $$\begin{array}{l} L_{2}\left(s|e\right)\propto S_{1}\left(e|s\right)\cdot p\left(s\right) \end{array}$$

Given the equations above, one may ask why the number of alternative states and expressions should play any role in the interpretation process. The reason is that the calculation of listener and speaker matrices *L*_0_(*s*|*e*), *S*_1_(*e*|*s*), and *L*_2_(*s*|*e*) presupposes a row normalization which, in case of listener matrices (5) and (9), has to take into account all possible states compatible with the uttered expressions *e*, and, in the case of the speaker matrix (8), all alternative expressions that the speaker could have used refer to *s*. Since we are going to analyze experimental responses from sliding scales, which are usually not normalized, this normalization procedure has to be applied to the experimental data, too. Such a procedure might affect the conclusions that are drawn with respect to an increase or decrease of probabilities *L*_2_(*s*|*e*), which is why we will also analyze unnormalized slider responses.

### 2.2. Narrow Focus in the RSA Framework

In this section, we will develop a model for the exhaustive inferences associated with prosodic focus as in expressions like (1-a) or the simpler (10). We will signal the narrow focus solely by capital letters that indicate prosodic stress in this paper.^4^

(10) BOB danced.

We only consider expressions in the context of a wh-question directly and congruently answered by them, such as (11-a) or (11-b), cf. Groenendijk and Stokhof (1984), Rooth (1992), and Beaver and Clark (2008). Thus, for all purposes of this paper, narrow focus is defined as the stressed constituent that answers the respective contextually given wh-question. Once the question is given, question-answer congruence alone will disambiguate focus. Hence, even if we did not write *Bob* in capitals in (10), knowing that (10) answers (11-a) would suffice to judge that *Bob* is the narrow focus and it should be prosodically prominent. The crucial difference between the two types questions in (11) is that in (11-a) the domain of relevant individuals the question refers to is left open for pragmatic interpretation, whereas in (11-b) the domain is made explicit either by enumeration or by anaphoric binding.

(11)
  a. Who danced?
  b. Which of Audrey, Bob, Dale danced?
    Which of them danced?
    Who danced, Audrey, Bob or Dale?

We study utterances like (10) in the context of domain explicit questions like (11-b) experimentally by presenting participants a particular focus expression uttered by a speaker as an answer to a wh-question and asking them about the probabilities that the speaker referred to the different possible states.^5^

In order to be able to apply the RSA model, we will need to fix a few parameters beforehand. In the following we will motivate and explicate the parameter settings that we use in this work. However, it is crucial to understand that our parameter choices have no bearing on the overall conclusions of the paper. To show this, in addition to presenting in section 2.3 the predictions our model makes given those choices, we also discuss the impact of alternative choices on model predictions in section 2.4.

Firstly, we consider the cost of an utterance *e*, *C*(*e*). A reasonable choice for *C*(*e*) would be to set it to values proportional to the number of words in the utterance which normally receive stress. However, we detected no influence of costs on the results in a pilot study with an experimental setup nearly identical to those in section 3 but where the number of words the names of all individuals consisted of was increased. Hence, at least the number of stressable syllables or the number of words does not seem to have any significant influence in our experimental design. Therefore, in this paper, we assume zero costs across the board for all expressions.

Secondly, we need to decide what states are considered by speakers and listeners. We consider only mutual exclusive partial states *s* which partition the set of possible worlds. The set *S* of partial states in the context of the question (11-b) is:

(12)
  $$\begin{array}{l} S:=\left\{s_{\emptyset },s_{\text{Audrey}},s_{\text{Bob}},s_{\text{Audrey}\&\text{Bob}},s_{\text{Dale}},\ldots \right\} \end{array}$$
  ^6^

Although one could assume a uniform prior distribution of states, we will determine *p*(*s*) experimentally.

The final component of RSA models, the lexicon L, contains all expressions that the speaker could use to communicate states to the listener. Besides prosodic narrow focus expressions, there are various expressions that could be included in L, like, for example:

(13)
  a. Only Bob danced.
  b. It was Bob who danced.
  c. Everybody danced.
  d. Nobody danced.

In order to determine which of these expressions are part of the lexicon considered by interlocutors, we performed a pilot experiment^7^ where we put participants into the role of the speaker who needs to communicate a certain state. As a result, nearly all participants chose prosodic focus expressions. This is especially true for domains consisting of less than 4 individuals, and even for larger ones, e.g., with domain size *k* = 8, there is a only a small group of participants who chose exclusives to communicate states. As we therefore assume that exclusives have very low salience and to keep our models as simple in design as possible, we will assume a simple lexicon consisting of focus expressions only. Within this lexicon, the set of alternative expressions is always equal to the number of alternative states, as given in (14), even though the literal meaning of those expressions does not actually coincide with any single one of the states, as clarified in Footnote 6:

(14) Alternative expressions:
  a. BOB danced
  b. DALE danced.
  c. BOB and DALE danced.
  d. ...

### 2.3. Model Predictions

If we define the exhaustivity *E*(*k*) of prosodic focus expressions at domain size *k* by the probability the listener assigns to the exhaustive state *s*_*exh*_, i.e., the state in which only the person who is mentioned in the focus expression executes the action in question,

(15)
  $$\begin{array}{l} E\left(k\right):=L_{2}\left(s_{exh}|e_{foc},k\right) \end{array}$$

and assume a) the lexicon L to contain only prosodic focus expression and b) the prior probabilities of states to follow a binomial distribution^8^, then the following convenient relation for exhaustivity holds:

(16)
  $$\begin{array}{c} E\left(k\right)=E{\left(2\right)}^{k-1}\\ E\left(2\right)=\frac{1}{1+{\left(1+p^{-\alpha }\right)}^{-1}\cdot \frac{p}{1-p}} \end{array}$$

Thereby, α is the decision noise parameter and *p* is the success probability of the binomial distribution. This relation is especially useful for computationally intensive cases. However, as we do not want to make strong assumption on probabilistic beliefs beforehand, we will use Equation (16) only for demonstration purposes. In the data analysis of the experiments, we compute full speaker and listener matrices.

**Figure 1:**
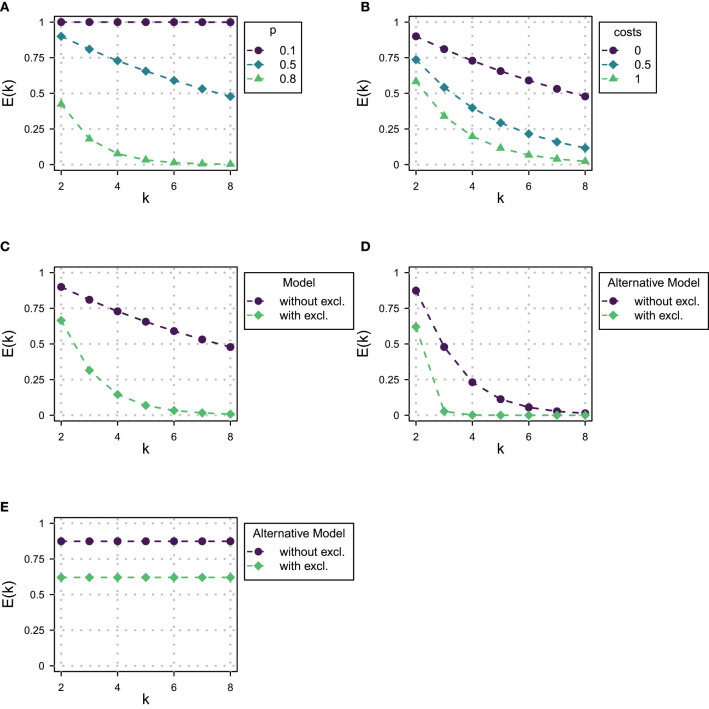
RSA model predictions for the Exhaustivity *E*(*k*). **(A)** For *p* ∈ {0.1, 0.5, 0.8} and α = 3. **(B)** For cost per word ∈ {0, 0.5, 1} with α = 3 and *p* = 0.5. **(C)** With and without exclusive alternatives with α = 3 and *p* = 0.5. **(D)** With and without exclusive alternatives with α = 3 and *p* = 0.5 and with 2 alternatives and 2 states. **(E)** With and without exclusive alternatives with α = 3 and with 2 alternatives, 2 states and constant prior probabilities.

To demonstrate the magnitude of the predicted effect of domain size on exhaustivity, Figure 1A shows the value of *E*(*k*) for different domain sizes and for different values of success probability *p* of the assumed binomial distribution of states. The exhaustivity of prosodic focus expressions drops dramatically with growing domain size, whereby the strength of the decrease gets stronger with larger values of *p*. Vice versa, for a fixed domain size, the model predicts a decrease in exhaustivity with increasing prior probability for non-exhaustive states, here manipulated by *p*. Although the probabilistic beliefs of participants might deviate from a binomial distribution, we expect that according to the RSA model outlined here a sufficient increase of domain size or prior probability of non-exhaustive states alone should lead to an observable drop in exhaustivity. This predicted decrease in exhaustivity is the core observation our paper is centered around.

### 2.4. Variations in the Model

Upon reflection, the predicted drop in exhaustivity with increasing domain size or increasing prior probability for non-exhaustive size seems rational. Indeed, at least statistically the posterior probability that only one specific person out of, say, three individuals present has a certain property must be higher than the probability that only this person has the property given four individuals, all other things being equal.

Intuitively, however, this prediction is surprising. In everyday communication we usually neither know nor care what the exact size of the domain is or what the prior probabilities are when we interpret answers to questions. Think of a simple example in (17). Usually, people who hear (17) will not ask themselves how many inhabitants the city under question has in order to process the answer.^9^

(17) A: Who makes more than a million a year in this city?
  B: SKYLAR.

Hence, while we acknowledge that the prediction of the RSA model needs empirical testing, we also want to avoid ascribing obviously false predictions to a theory that could be avoided within the usual logic of the framework. So, it is natural to ask whether the predicted dependency of exhaustivity is an artifact of the particular way in which we have applied the framework to the phenomena in question or whether it is an essential feature of the way the RSA framework handles such scenarios. In order to show that, indeed, this prediction is a general feature of the RSA framework, we will consider some alternative ways to set up the model and explore their predictions on the dependency of exhaustivity on domain size. In a first step, in section 2.4.1, we thereby consider the impact of each parameter of the model in isolation thus showing that none of them has the potential to change the essential characteristics of the predictions. In a second step, in section 2.4.2 we consider some more radical measures reflecting the possibility that interlocutors may in fact not use the entire space of alternatives or states when computing exhaustivity.

#### 2.4.1. Alternative Parameters

We have set the cost parameter to 0 for all expressions. Do the predictions of the model change when we include costs for longer statements? In Figure 1B we show the impact of including costs per word in the answer. By cost per word, we mean including a cost factor for every word within the narrow focus domain, for all other words in the answer remain constant across domains. It turns out that increasing costs will do exactly the opposite and increase the drop of exhaustivity with increasing domain size. This is because now the listener may reason that the speaker did not enumerate all alternatives which have the property under question because it would have been too costly. Besides fixing costs to 0, we have not included exclusives as alternatives in the model that we will use in the paper on the grounds that we observed that participants in pilot experiments mainly only used narrow focus constructions to answer the wh-question. But including exclusives as an alternative to the narrow focus construction would not remove the drop in exhaustivity, either: in fact, as shown in Figure 1C, the exhaustivity drops even stronger once exclusives are included into the model.^10^ We conclude that changing the parameters we have assumed for the RSA model in the previous section would in no way change the general predictions of the model, since including costs and exclusives would lead to an even steeper decline of predicted exhaustivity with increasing domain size.

#### 2.4.2. Alternative Models

Another important question is whether it is reasonable to assume that interlocutors actually use the multitude of states introduced by the question and the focus alternatives in the process of meaning computation. Here is one alternative possibility: The Gricean listener may have only two states and only two alternatives in mind. In particular, for the dialogue in (18), we would get the alternatives in (19). Of course, this is limited to those alternatives that are in fact compatible with the meaning of the actual answer.

(18)
  a. Q: Who danced, A, B or C?
  b. Answer: A danced.
(19)
  a. States:
    (i) Exhaustive State: *s*_*exh*_ : A danced and B and C did not.
    (ii) Non-Exhaustive State: *s*_*nonexh*_: A danced and B or C danced.
  b. Alternative expressions: { A danced. A danced and B or C danced. }

While one might intuitively imagine that reducing the actual number of states and alternatives the interlocutors reason about would potentially get rid of the problem, it turns out, as shown in Figure 1D, that the drop in exhaustivity is in fact increased rather than decreased by this measure and adding exclusives to the set of alternatives leads to an even steeper curve. The main reason is that in the RSA-logic reducing the number of alternatives in the way described means putting all non-exhaustive alternatives together, so the corresponding probabilities do not vanish but accumulate in this one alternative. A different approach that comes to mind is the model of Degen et al. (2015) for scalar implicatures. This approach protects against extreme values of prior probabilities by assuming listeners to use a uniform back-off prior. However, since an increment in domain size introduces more alternatives, a uniform back-off prior still reduces the prior probability of the single exhaustive state. Therefore, such a model would be ineffective against variation of domain size.^11^

One type of RSA-model that would avoid the prediction that exhaustivity drops would be one in which both the number of states and alternatives were reduced to two and the prior probability of the non-exhaustive state would also be assumed not to drop with the increasing number of alternatives. The predictions of such a variant of the model are given in Figure 1E. However, it should be noted that assuming the same prior for the exhaustive and the non-exhaustive state in the case of larger numbers of alternatives is deeply incompatible with the basics of probability calculus and goes way beyond accommodating incomplete or imperfect handling of probabilities by interlocutors. In fact, such a model devoid of probabilistic dependencies can hardly be called an implementation of Bayesian inference.

This leads us to the conclusion that the RSA model combined with the usual understanding of focus and question semantics predicts a decreasing level of exhaustivity as the number of alternatives increases. If this is empirically borne out, it constitutes very strong evidence for the model in general. However, if not, it is really hard to see how one can use a classical RSA model to handle focus exhaustivity, thus raising the question what model could be a reasonable alternative and why the RSA model, albeit successful in other domains, would fail in the case of narrow focus exhaustivity.

## 3. Experiments

We performed two experiments where we collected probability judgements on sliding scales in order to determine whether the RSA model predictions outlined in the last section are correct and exhaustivity indeed decreases with growing domain size.

Both experiments are parted in two subexperiments, one for elicitation of prior probabilities *p*(*s*) and one for elicitation of posterior probability *L*_2_(*s*|*e*_*foc*_, *k*) of the possible states *s* ∈ *S*. Since the RSA model provides predictions for posterior probabilities based on prior probabilities, both quantities need to be measured in order to test model predictions for exhaustivity *E*(*k*) of narrow focus expression *e*_*foc*_.

The main difference between both experiments is in the way alternative states are presented by sliders: In Experiment 1, one slider for each state was presented in the items. As will be shown, this setup entails some conceptual difficulties concerning the interpretation of the results. We therefore conducted a second experiment where all alternative non-exhaustive states are condensed into one state description and are therefore represented by one slider. In addition, in Experiment 2 we tested the general dependency of exhaustivity on prior probability in additional trials with a fixed domain size.

### 3.1. Experiment 1

#### 3.1.1. Participants, Materials and Procedures

109 participants with English as first language^12^ were recruited through *Prolific Academic* (www.prolific.ac) and responses for 18 different items were collected in return for a small payment. Thirteen participants where excluded because they took less than 15 s on at least one of the three items or they assigned the value 0 to all states.

**Figure 2:**
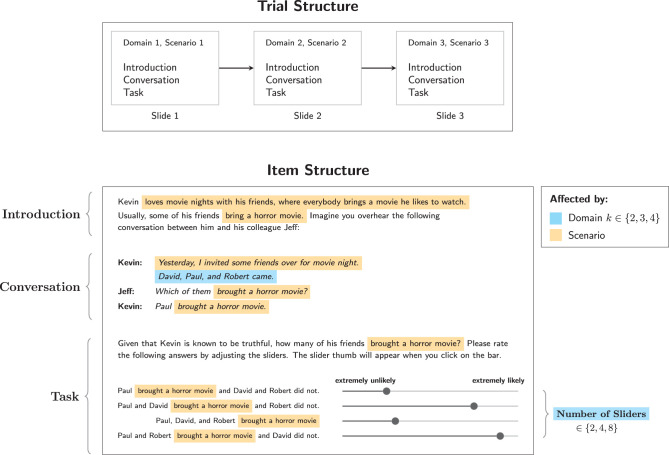
Structure of trials in Experiment 1. Also shown is the structure of items for posterior elicitation, where the elements that are affected by the choice of domain size and scenario are highlighted.

Figure 2 illustrates the structure of trials. Every participant saw three items, each for a different domain size and scenario, where a scenario is a combination of a regularly occurring event and some specific action (target action). The domain sizes used were *k* ∈ {2, 3, 4}. Table 1 gives an overview of the elements which were combined to form the items.

**Table 1:** Possible combination of elements used in the items for Experiment 1.

**Scenarios**		**Probability type (n_part_)**	**Domain sizes**
**Occasion**	**Target action**		
Being on vacation Halloween dinner party Movie night	Going camping Dressing up as a superhero Bringing a horror movie	Prior probability (30) Posterior probability (66)	*k* ∈ {2, 3, 4}

Figure 2 also illustrates the structure of items for posterior probability elicitation, which were always composed of three parts: An introduction, a conversation, and the task, all presented on one slide. The introduction introduced a person named Kevin in connection with the description of some regularly occuring occasion. The occasions used in the three different scenarios were:

(20)
  a. Being on vacation (Scenario 1)
  b. Halloween dinner party (Scenario 2)
  c. Movie night (Scenario 3)

In the introduction it was also stated that some of Kevin's friends usually perform some specific action (target action) on these occasions. We used the wording “usually, some of his friends Y'ed,” with “Y” being the target action, as we wanted the probability for Kevin's friends to perform that action to be somewhere in the center of the scale. The target actions corresponding to the three scenarios in (20) were:

(21)
  a. Going camping (Scenario 1)
  b. Dressing up as a superhero (Scenario 2)
  c. Bringing a horror movie (Scenario 3)

The introduction was followed by a conversation between Kevin and his colleague Jeff, which was presented to participants in a written form. It consisted of a) a statement made by Kevin, b) a question posed by Jeff, and c) an answer given by Kevin. Kevin's statement describes a concrete instance of the regularly occuring event and gives a list of friends that were present. The latter provides participants with the necessary information about the domain size. For every item, this list was obtained by drawing randomly from a list of frequent English first names, which were then ordered alphabetically in order to reduce the cognitive load of the task. Jeff then asks which of Kevin's friends performed the target action. This is followed by Kevin's answer, which was always of the form of a narrow focus construction “Y X'ed” where “Y” was only one of the friends mentioned before.^13^ Participants were then asked to evaluate the probability of all different combinations of friends performing the respective action on the occasion, corresponding to the posterior probability of the different states. Participants indicated their probability judgements by moving a set of sliders whose order was randomized. See Supplementary Figure 4 for an example item of posterior elicitation.

We omitted indication of specific prosody in the narrow focus expression used as answer in the conversation. This is because on the one hand, we only expect trained linguists to interpret formal indications of prosody correctly. On the other hand, we are confident that participants read the answer in the majority of cases with the “correct” focus prosody, as it is clearly related to the preceding question, as discussed in section 2.2.^14^

Note that the number of possible states varies with domain size, so the number of sliders shown varies, too, as indicated in Figure 2. In posterior elicitation items, for *k* = 2, there are 2 possible states, corresponding to 2 sliders. For *k* = 3, 4 sliders were shown, and for *k* = 4, 8 sliders were shown.

Items for prior probability elicitation consisted of only two parts: (a) An introduction where a concrete instance of the regularly occurring occasion was described including the list of friends present, and (b) the task, where participants were asked to evaluate the probability of all different combinations of friends performing the target action, corresponding to the prior probability of the different states. See Supplementary Figure 3 for an example item. As with posterior elicitation items, the number of sliders shown varied with domain size: For *k* = 2, there were 4 possible states, corresponding to 4 sliders. For *k* = 3, 8 sliders were shown, and for *k* = 4, 16 sliders were shown.

30 participants received items in which prior probabilities were elicited, while the rest (66 participants) saw items where posterior probabilities were elicited.^15^

#### 3.1.2. Data Modeling and Analysis

**Figure 3:**
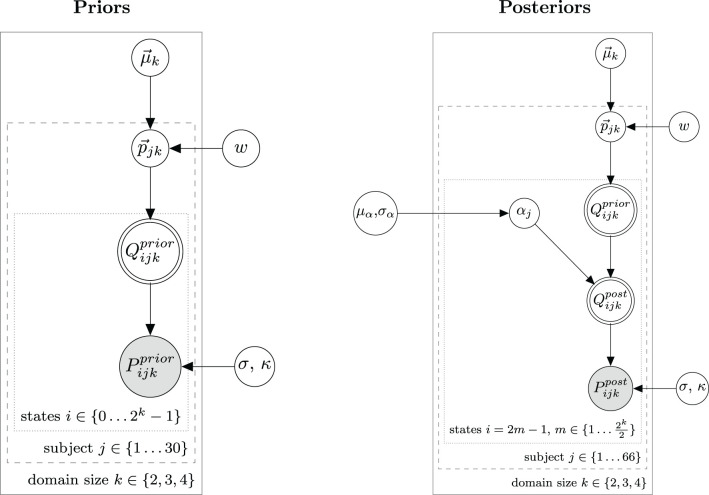
Bayesian graphical models for prior probabilities (left) and posterior probabilities (right) used for data analysis in Experiment 1.

We use a hierarchical Bayesian model to model the distribution of prior and posterior probabilities. As dependent measure we use normalized slider ratings. For the elicitation of prior beliefs, our model is practically identical to one used for slider rating data in Franke et al. (2016). For posterior beliefs, the RSA specific part was added to the model, which establishes the link between prior and posterior probabilities. Figure 3 shows the hierarchical Bayesian models for prior and posterior probabilities with parameters as defined by Equation (22).

(22)
  $$\begin{array}{l} \kappa \sim Gamma\left(5,5\right)\\ w\sim Gamma\left(2,0.1\right)\\ \sigma^{2}\sim inv.Gamma\left(1,1\right)\\ {\vec{\mu }}_{k}\sim \text{Dirichlet}\left(\left[1,\ldots ,1\right]\right)\\ {\vec{p}}_{jk}\sim \text{Dirichlet}\left(w\cdot {\vec{\mu }}_{k}\right)\\ Q_{ijk}^{prior}=\frac{p_{j,m_{i}+1}}{\left(\frac{k}{m_{i}}\right)},m_{i}=|B_{i}|\\ logit\left(P_{ijk}^{prior}\right)\sim \text{Norm}\left(logit\left(Q_{ijk}^{prior},\kappa \right),\sigma \right) \end{array}$$

The target set *B*_*i*_ refers to the subset of the domain corresponding to the state *s*_*i*_ where only the individuals in this subset perform the target action. For computational purposes, we enumerate the states following the binary encoding scheme exemplified in Table 2 for the domain of the question (11-b). Since all subsets *B*_*i*_⊂{Audrey, Bob, Dale} can be ordered and enumerated by binary numbers, we number the states by the decimal number obtained from the binary number *i* of their corresponding subset *B*_*i*_.

**Table 2:** Binary encoding of subsets of individuals for the domain consisting of {Audrey, Bob, Dale}.

**State number i**	**Binary number**	**B_i_**
0	000	∅
1	001	{A}
2	010	{B}
3	011	{A,B}
4	100	{D}
5	101	{A,D}
6	110	{B,D}
7	111	{A,B,D}

*Names were abbreviated by their first letter*.

Like the model in Franke et al. (2016), we model peoples beliefs on prior probabilities ${\vec{p}}_{jk}$ as being Dirichlet distributed among the population with means ${\vec{\mu }}_{k}$ and a concentration parameter *w* which controls the size of the variance. κ is a parameter of the logit transformation and controls for endpoint aversion or affinity of participants responses. The hyperparameter of means ${\vec{\mu }}_{k}$ is generated from a flat Dirichlet distribution.

It is important to note that the vector ${\vec{p}}_{jk}$ is not consisting of 2^*k*^ entries for the whole state space. This is because we assume that all states with the same size of *B*_*i*_ are equally probable since they are conceptually indistinguishable.^16^ This has the convenient consequence that the number of independent hyperparameters ${\vec{\mu }}_{k}$ grows only linearly with domain size, with one parameter for every possible size of the target set. So for *k* ∈ {2, 3, 4}, ${\vec{\mu }}_{k}$ has lengths of 3, 4, and 5, respectively. From the individual beliefs ${\vec{p}}_{jk}$ on the probability of target set sizes the probability vector for all states ${\vec{Q}}_{jk}^{prior}$ is obtained by redistributing every entry of ${\vec{p}}_{jk}$ equally over all states compatible with that target set size.

The corresponding Bayesian model for posterior probabilities is shown in Figure 3 right and definitions of model parameters not already defined in Equation (22) are given in Equation (23).

(23)
  $$\begin{array}{l} \mu_{\alpha }\sim Gamma\left(5,5\right)\\ \sigma_{\alpha }^{2}\sim inv.Gamma\left(1,1\right)\\ \alpha_{j}\sim \text{Gamma}\left(\frac{\mu_{\alpha }^{2}}{\sigma_{\alpha }^{2}},\frac{\mu_{\alpha }}{\sigma_{\alpha }^{2}}\right)\\ Q_{ijk}^{post}\propto S_{1}\left(foc|i\right)\cdot Q_{ijk}^{prior}\\ S_{1}\left(foc|i\right)\propto e^{\alpha \cdot U\left(foc,i\right)}\\ U\left(foc,i\right)=log\left(L_{0}\left(i|foc\right)\right)\\ logit\left(P_{ijk}^{post}\right)\sim \text{Norm}\left(logit\left(Q_{ijk}^{post},\kappa \right),\sigma \right) \end{array}$$

*L*_0_ is as defined in Equations (5, 6) and *foc* designates the focus expression used by the speaker in the items. $Q_{ijk}^{prior}$ was drawn in the same way as in Equation (22), but now hyperparameters *w* and ${\vec{\mu }}_{k}$ were set to the means of their posterior distributions obtained by fitting the model for prior probabilities.

$Q_{ijk}^{post}$, contrary to $Q_{ijk}^{prior}$, contains only states compatible with the uttered expression, which comprise half of the full state space of the prior probabilities, i.e., all states where at least the individual mentioned in the expression is part of the target set. This Model, in addition to the elements of the prior model, contains as a central element the computation of posterior beliefs $Q_{ijk}^{post}$ from prior beliefs $Q_{ijk}^{prior}$. This RSA specific part is where the model defined in sections 2.1, 2.2 is used in order to model posterior beliefs as the interpretations of a Gricean *L*_2_ listener.

As laid out in section 2.1 the RSA model also depends on the rationality parameter α. We take α to be gamma-distributed among the population with mean μ_α_ and standard deviation σ_α_.^17^ To remain relatively uncommitted concerning the hyperprior μ_α_ we used a weakly informative Gamma distribution with mean 1 and a higher probability for values in the vicinity of 1.^18^ $\sigma_{\alpha }^{2}$ models the variance of α and is drawn from an inverse Gamma distribution.

One advantage of having an individual based α is that it also covers possible spillover effects between items.^19^ So, for example, if a participant chooses higher or lower slider values for the exhaustive state consistently for all three items, this is captured by the model assigning more probability to a larger or lower α for this participant, respectively.^20^

For prior and posterior probabilities we used Stan (Carpenter et al., 2017) to collect samples from the joint posterior distribution. We used 4 chains with a warm up phase set to 50% of total iterations. The total number of iterations was always 50, 000, the thinning factor was five. We ensured that scale reduction factors were close to one and that no divergent transitions occurred during simulation.

#### 3.1.3. Results: Prior Probabilities

**Figure 4:**
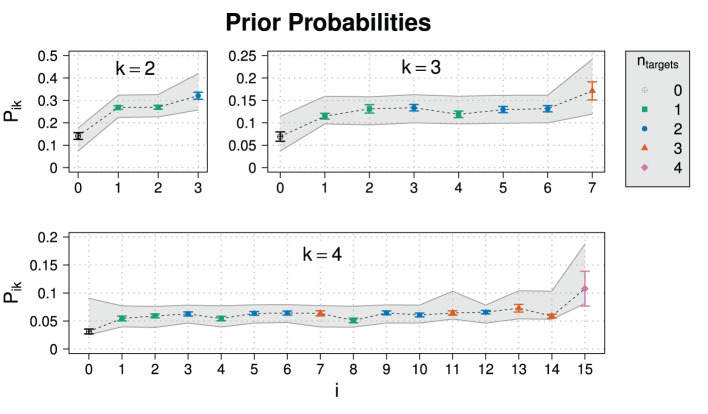
Experimental results for the prior probability for normalized slider values *P*_*ik*_ for different states *i* and domain sizes *k* in Experiment 1. Also shown are posterior predictive 95% HDIs (gray areas).

Figure 4 shows the results for the prior probabilities. As an ANOVA revealed that there was no significant dependence on the scenario, we collapsed over the three scenarios.^21^ The gray areas are 95% high-density intervals (HDIs) for the population average calculated by drawing 20,000 samples from the posterior predictive distribution. Means and 95% HDIs of the posteriors for model parameters are in Supplementary Table 1.

The model predictions on prior probabilities suggest that the model can cope well with the data. The value for the parameter κ is close to one, indicating that normalized slider values map approximately linearly to prior beliefs.

To test the compatibility of the model with individual level data, we calculated posterior predictive *p*-values (Gelman et al., 2014) using the individual responses for exhaustivity in every item as test statistic. 4.6% of the responses resulted in *p*-values of *p* < 0.05 for Model, which suggests that the model fits the data well on individual level, too.

**Figure 5:**
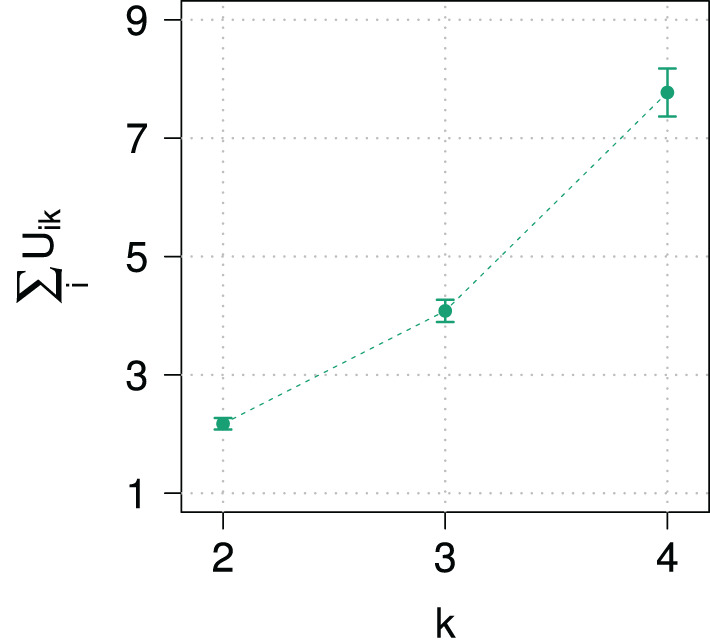
Average sums of unnormalized slider values *U*_*ik*_ for prior probabilities in Experiment 1.

As already mentioned in section 2.1, responses from sliding scales have to normalized for the analysis. It therefore seems natural to also check the unnormalized responses. In order to see how participants distributed probability over the different sliders at different domain sizes, Figure 5 shows the sum of unnormalized slider values, which ideally would be close to one or at least approximately constant for all domain sizes, indicating that participant normalize their slider ratings consistently. However, the sum exceeds 1 and increases strongly with domain size, implying that participants did not normalize their responses, at least not in a consistent way.

#### 3.1.4. Results: Posterior Probabilities

**Figure 6:**
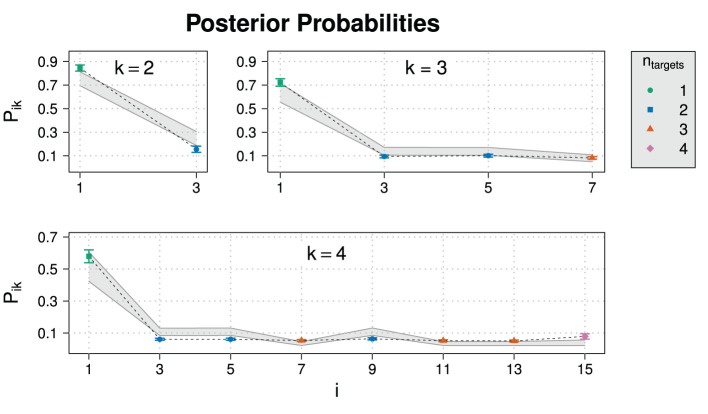
Experimental results for the posterior probability for normalized slider values *P*_*ik*_ for different states *i* and domain sizes *k* in Experiment 1. Also shown are posterior predictive 95% HDIs (gray areas).

**Figure 7:**
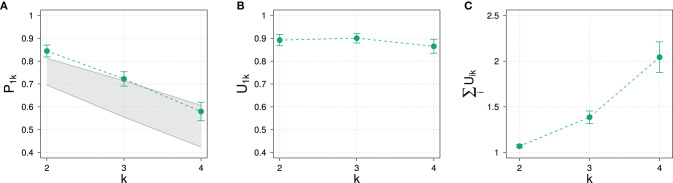
**(A)** Normalized and **(B)** unnormalized slider values for the exhaustive state *i* = 1 as well as **(C)** average sums of raw slider values in Experiment 1.

Figure 6 shows the posterior probabilities of normalized slider values for Experiment 1. Figure 7A repeats the corresponding values and additional statistics for the exhaustive state *i* = 1 only. Also shown are 95% HDIs of posterior predictive samples.^22^ Means and 95% HDIs of the posteriors for model parameters are in Supplementary Table 2. An ANOVA revealed that the influence of the domain size on *E*(*k*) is highly significant (*p* = 5.35·10^−8^) for normalized slider values but not for unnormalized slider values (*p* = 0.457).

The comparison between the experimental data with the 95% HDIs suggests that the model reproduces the trend of a dropping exhaustivity, although most data points are not inside HDIs. Concerning the non-exhaustive states, the model is not able to reproduce their posterior probability well: While the RSA model predicts fine grained differences between non-exhaustive states, the empirical data suggests that participants on average did not discern between those states. This discrepancy between model and data is more pronounced at larger domain sizes.

As this suggests some kind of mismatch between the model and the way participates respond, we additionally show unnormalized slider values and average sums of unnormalized slider values in Figures 7B,C. As can be easily observed, participants chose on average approximately the same unnormalized slider value for the exhaustive state across all domain sizes. As observed with prior probabilities, the average sum of unnormalized slider values increases for posterior probabilities, too. This means that the drop of exhaustivity is produced during normalization of the raw slider values: Although the raw slider value for the exhaustive state is approximately constant across all domain sizes, during normalization it is divided by the sum of all raw sliders values, which, in contrast, increases with domain size due to the increasing number of sliders.

#### 3.1.5. Discussion

The analysis of the prior probabilities revealed a good fit between the model for prior probabilities, but also strong deviations of raw slider values from normalized slider values.

The same kind of deviation was also found in the results for the posterior probabilities. On average, participants did not change slider positions for the states across domain sizes, while the sum of unnormalized slider values grew with domain size.

The model reproduces the observed trend of a decrease in exhaustivity, but is not able to fit most data points satisfactory. Especially for non-exhaustive states there is a pronounced mismatch between model and observation: We do not observe differences between the non-exhaustive states as predicted by the model.

We do not wish to judge the imperfect performance of the model too harshly. On first sight, one could certainly highlight that all in all the model reproduces the trend of a decreasing exhaustivity which we observed experimentally. Notwithstanding, interpreting this as a clear success feels premature given the mismatch between raw and normalized data. This mismatch raises the suspicion that the observed exhaustivity is actually an artifact of design produced by presenting participants a growing number of sliders. As was already pointed out, participants gave approximately constant raw slider ratings, so the decrease in exhaustivity emerges during normalization, where the approximately same raw slider value for the exhaustive state is divided by an increasing raw probability mass generated by the growing number of sliders. So the natural concern is that we possibly cannot trust the normalized slider values.

This calls the experimental design into question. Do constant raw slider ratings reflect constant probabilistic beliefs or just an inability to normalize the sliders correctly? It is conceivable that participants did not actually believe that the probability of exhaustivity decreases with increasing domain size at all. Additionally, if this is the case, interpretation of narrow focus expressions in the experiment would not only be insensitive to information on domain size but also to prior probabilities of the different states, since participants judged prior probabilities to change with domain size. This possibility raises the question whether participants take prior probabilities into account at all. In order to shed light on these questions we conducted a further experiment, to be described below.

### 3.2. Experiment 2

#### 3.2.1. Participants, Materials and Procedures

We recruited 382 participants through *Prolific Academic*. None of them had participated in the previous experiments. Thirty-five participants where excluded because they took less than 15 s on at least one of the three items or they assigned the value 0 to all states. Scenarios, items and general design were identical to those of Experiment 1. The crucial difference is that we now used a fixed number of two sliders for all domain sizes, corresponding to the following two state descriptions:

(24)
  a. X Y'ed and the other friends did not.
  b. X and at least one of the other *k* friends Y'ed.

**Figure 8:**
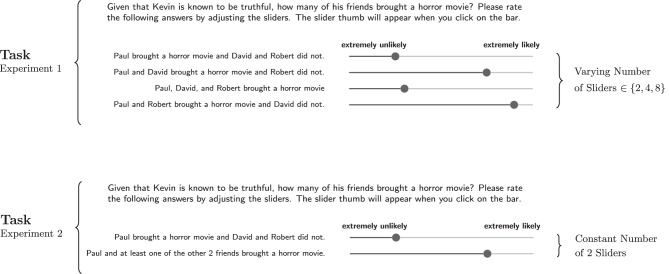
Changes in the number of presented sliders in tasks of Experiment 2 in comparison to Experiment 1.

The resulting changes in task structure are illustrated in Figure 8 in comparison to the task part of Experiment 1.

The reason for this change in design is that we intended to separate effects associated with normalization of subjective probabilistic beliefs from a linguistic concept of exhaustivity, i.e., we want to know if participants actually believe and interpret focus expressions to be less exhaustive at larger domain sizes, not if focus expressions are less exhaustive from an abstract and possibly normative point of view. So even if there is a non-trivial effect of normalization, presenting all items with a binary partition of the state space like (24) should ameliorate normalization effects and render responses for different domain sizes comparable.

As in Experiment 1, one part of the participants (142 participants) was asked to evaluate prior probabilities, while the other part (205 participants) evaluated posterior probabilities. See Supplementary Figures 5, 6 for example items.

An additional difference is that the number of sliders in prior and posterior probability elicitation items is now identical, too. In order to achieve this, when eliciting prior probabilities, participants were given the information that one person of the domain size already performed the action in question, thus rendering the state space identical to the one in items of posterior probability elicitation.^23^ This way we hope to remove potential effects resulting from a mismatch between the presentation of alternatives in prior and posterior elicitation. Table 3 gives an overview of the elements which were combined to form the items in Experiment 2.^24^

**Table 3:** Possible combination of elements used in the items for Experiment 2.

**Prior type**	**Scenarios**	**Probability type (n_part_)**	**Domain sizes**	**Items per part**.
“none”	being on vacation halloween dinner party movie night	prior probability (49) posterior probability (82)	*k* ∈ {2}	1
“some”	being on vacation halloween dinner party movie night	prior probability (65) posterior probability (60)	*k* ∈ {2, 3, 4}	3
“all”	being on vacation halloween dinner party movie night	prior probability (28) posterior probability (63)	*k* ∈ {2}	1

In order to check whether prior probabilities influence participant's decisions regarding posterior probabilities at all, we conducted additional trials which tested two different kinds of priors. This time, the domain size was fixed at *k* = 2, so participants of these special trials only received one item instead of three. In these two conditions, we replaced the wording “Usually, some of his friends [...]” by “Usually, all of his friends [...]” and “Usually, none of his friends [...],” respectively. Both wordings should elicit prior probabilities different from the ones elicited by the original wording: The “all” prior should eliciting lower probability of the exhaustive state, while the “none” prior should elicit higher probability.

We chose the fixed domain size *k* = 2 for three reasons: First, the prior and posterior probability computations here almost coincide with the ones of Experiment 1, which gives us an additional test of the data and results. Second, the number of alternative non-exhaustive states at this domain size is just one. That way, we avoid conceptual problems regarding the distribution of probability across the different alternative states, to explained below, so the measured normalized slider values at this domain size can be identified with the probabilities of the corresponding states.

Third, as there are only two alternative states, the results are more generalizable and can be used as a test of the general applicability of Bayes' theorem

(25)
  $$\begin{array}{l} p\left(s|e\right)=\frac{p\left(e|s\right)\cdot p\left(s\right)}{\sum_{s^{\prime }}p\left(e|s^{\prime }\right)\cdot p\left(s^{\prime }\right)} \end{array}$$

to the phenomenon of focus exhaustivity, also underlying the more specific RSA formulation Equation (9). To see why the results can be generalized in such a way, consider that at *k* = 2 the following holds:

1. There are only two possible states after a speaker uttered *foc*_*A*_, i.e., *s*_*A*_ and *s*_*AB*_. Therefore, *p*(*s*_*AB*_) = 1 − *p*(*s*_*A*_).
2. If *s*_*A*_ is the actual state, speakers must utter *foc*_*A*_, so *p*(*foc*_*A*_|*s*_*A*_) = 1, since *foc*_*AB*_ would be a false statement.

Using this as well as setting *s* = *s*_*A*_ and *e* = *foc*_*A*_, Equation (25) becomes

(26)
  $$\begin{array}{l} p\left(s_{A}|foc_{A}\right)=\frac{1}{1+p\left(foc_{A}|s_{AB}\right)\cdot \frac{1-p\left(s_{A}\right)}{p\left(s_{A}\right)}} \end{array}$$

This means that if we assume that

(27) the speaker function *p*(*foc*_*A*_|*s*_*AB*_) is a monotonically decreasing function of *p*(*s*_*A*_)

then *p*(*s*_*A*_|*foc*_*A*_) is a strictly increasing function of *p*(*s*_*A*_). So under Condition (27), increasing or decreasing the prior probability *p*(*s*_*A*_) should lead to an increase or decrease in exhaustivity *p*(*s*_*A*_|*foc*_*A*_) of a focus expression *foc*_*A*_ independently of other model assumptions besides (27) and Bayes' theorem.^25^

Equation (26) also implies that for *p*(*s*_*A*_) = 0 and *p*(*s*_*A*_) = 1, posterior beliefs should reach *p*(*s*_*A*_|*foc*_*A*_) = 0 and *p*(*s*_*A*_|*foc*_*A*_) = 1, respectively. Consequently, if the variation of the prior covers a large enough portion of the interval [0, 1], we should expect to observe a variation in exhaustivity under all models that assume (27) and are based on Bayes' theorem, including the RSA model. We test this prediction by varying the prior at *k* = 2.

#### 3.2.2. Data Modeling and Analysis

For the analysis of this experiment we refrained from fitting any specific models to the data for two reasons. First, we are primarily interested in the question whether exhaustivity is affected by a) the number of presented sliders or by b) prior probabilities. Before we are able to devise a specific model for the phenomena in question, this conceptual question needs to be addressed first.

Second, the design of this experiment complicates the mapping of sliders to the probabilities of their corresponding states: For the domain sizes *k* ∈ {3, 4} we now measure sums over probabilities of non-exhaustive states, not the probabilities of the individual states. Since there are many possible mappings between the sum over states and their individual values, we cannot straightforwardly derive the individual values needed for the computation of the speaker and listener matrices.

Nonetheless, for domain size *k* = 2, we will calculate approximate predictions for posteriors based on the priors measured in this experiment and by using the values of the parameters α and σ from Experiment 1.^26^ This way, we hope to get at least a rough idea of the magnitude of effects one would expect if using an RSA model similar to the one of Experiment 1.

#### 3.2.3. Results: Prior Probabilities

**Figure 9:**
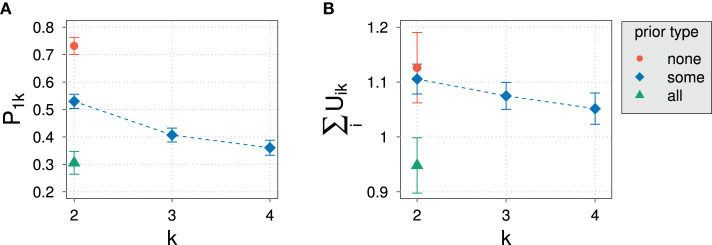
Experimental results for the prior probability for normalized slider values *P*_1*k*_ **(A)** and for average sums of unnormalized slider values **(B)** for different domain sizes *k* and prior types in Experiment 2.

Figure 9 gives the results of the experiment for normalized slider values of the different states for different domain sizes and prior types. Also shown are the sum of unnormalized slider values for the “some” prior condition.

In comparison with Experiment 1, the decrease of prior probability with domain size in Experiment 2 is less steep, comprising a difference of only about 20%. The variation in prior type, on the other hand, had the expected effect of substantially increasing and decreasing the elicited prior probability: The prior probability increases and decreases by about 20% depending on the prior. This means that we indeed cover a significant portion of the possible range of prior values. Based on the model parameters resulting from the model fit of Experiment 1, we can roughly estimate the posterior probability that one would expect according to the RSA model: The exhaustivity for “some,” “none,” and “all” priors at *k* = 2 is expected to be at approximately 85, 95, and 61%, respectively. We expect such a variation to be clearly visible in the data.

Also unlike in Experiment 1, the average sum of unnormalized slider values is always close to 1 and stays approximately constant across domain sizes. The kind of prior seems to influence the sum of slider values only to a small degree compared to the variation of sums observed in Experiment 1.

The constancy of average sum of unnormalized slider values already indicates an impact of the design on experimental results: Although the set of non-exhaustive states grows with domain size like in Experiment 1, participants now assign approximately the same total probability mass to all states.

#### 3.2.4. Results: Posterior Probabilities

**Figure 10:**
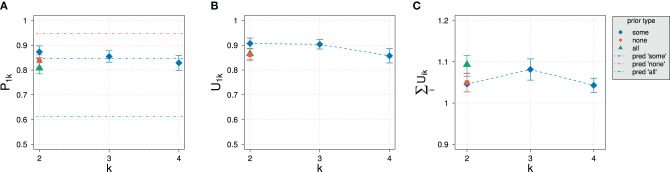
Experimental results in Experiment 2 for different domain sizes *k* and prior types. **(A)** Posterior probability for normalized slider values *P*_1*k*_. Also indicated are approximate predictions for the exhaustivity for *k* = 2. **(B)** Unnormalized slider values *U*_1*k*_. **(C)** Average sums of unnormalized slider values.

An ANOVA revealed that there is no dependence on the type of scenario of normalized slider values, so we collapsed over the three scenarios. Figure 10 shows the results for the elicitation of posterior probability for the normalized and unnormalized slider values for the exhaustive state *i* = 1 an for the average sum of slider values.

As observed with elicitation of prior probabilities, the average sum of unnormalized slider values stays approximately the same, exceeding 1 only slightly. In strong contrast to Experiment 1, the influence of domain size on normalized and unnormalized slider values is not significant (*p* = 0.227 and *p* = 0.138, respectively). Even more surprising, the different prior types do not seem to have a significant effect: An ANOVA revealed that there was no significant variation in exhaustivity (*p* = 0.07). Although there might possibly be a small effect, based on RSA predictions we would have expected that the exhaustivity is at about 95 and 61% for the “none” and “some” prior type, respectively. Only the posterior for the “some” condition at *k* = 2 is as expected.

The principal commonality with the results of Experiment 1 is the constancy of unnormalized slider values for the exhaustive state. In both experiments, the value is about 0.9 for all domain sizes. The use of a different prior did not have any significant effect on the raw slider values (*p* = 0.257), either.

#### 3.2.5. Discussion

To summarize the results of Experiment 2, we observe

1. in contrast to Experiment 1, constant average sums of raw slider values
2. like in Experiment 1, constant unnormalized slider values for all domain sizes
3. in sharp contrast to Experiment 1, constant normalized slider values for the exhaustive state at all domain sizes
4. no significant effects of the variation of prior probability on exhaustivity.

The constant average sum of slider values indicates an impact of the experimental design on the results: Since in Experiment 2 variation in domain size did not influence the average sum of unnormalized slider values, the variation of the number of presented sliders in Experiment 1 seems to influence participants responses. This strengthens our suspicion that the results of Experiment 1 cannot be interpreted straightforwardly as a confirmation of the predictions of the RSA model, due to the non-trivial influence of experimental design and normalization procedure.

At the same time, as observed in Experiment 1, unnormalized slider values are constant on average across all domain sizes, suggesting that on average participants might believe the probability of the exhaustive state to be approximately constant.

The most crucial results, however, are the absence of an exhaustivity drop as observed in Experiment 1 and the ineffectiveness of variation of the prior probability. Although we suspected that the results of Experiment 1 were influenced by experimental design, we did not expect them to disappear completely. If alternative states and their corresponding probabilities are in one way or the other part of the processes underlying the interpretation of expressions, a growing number of non-exhaustive states should influence posterior beliefs after all.

Even though one could try to explain the constancy of exhaustivity in the “some” condition by recourse to the now weaker drop in prior probability across different domain sizes, the absence of a significant effect of direct variation of the prior probability poses a deep and persisting conceptual problem: At *k* = 2 there is only one non-exhaustive state which therefore must be salient. Still, at this domain size, the observed exhaustivity for “none” and “all” prior is very far from where one would expect it to be. As already argued, the variation in prior probability elicited at this domain size should lead to a variation in exhaustivity on any Bayesian account of a rational speaker. Instead, we observe a clustering of posteriors for all three priors at around the same value in the range of 80 − 86%. Since we used a rather large number of participants, this is unlikely to be the result of a statistical error, especially considering that we reproduce the same exhaustivity value for *k* = 2 as in Experiment 1.

It appears that, for at least the majority of participants, prior probabilities of states play, if at all, only a minor role in the process of meaning computation. Even if there are small effects of the prior, we do not know of any way to account for the obtained results in terms of a model in terms of reasoning based on (25) and (27).

In short, Experiment 2 confirms our suspicion that the decrease in exhaustivity observed in Experiment 1 is due to experimental design and provides further evidence for the possibility that exhaustivity might in fact be constant or approximately constant across domain sizes and different priors, contrary the predictions of the RSA Model. Furthermore, the results question the general possibility of modeling focus exhaustivity by Bayesian inference based on probabilities of states.

## 4. General Discussion and Conclusions

We have shown above that the RSA model outlined should predict a decrease of exhaustivity of narrow focus constructions with increasing domain size and with increasing prior probability of non-exhaustive states, for a wide range of parameter settings and conceptual design choices. In order to see whether this prediction is borne out, we have conducted two experiments that in sum provide strong evidence against the model.

In Experiment 1 we have asked participants to rate the exhaustivity of narrow-focus constructions using one slider for each semantically admissible state, hence the number of sliders increased with domain size. The fitted RSA model indeed predicts a decrease in exhaustivity which the data approximately follows. However, this success could not unequivocally be attributed to the model because besides the fit being far from completely satisfactory we observed a mismatch between decreasing normalized slider values and constant raw slider values.

This raised two conceptual concerns:

1. Do normalized or unnormalized slider values reflect participant's probabilistic beliefs? Or, in other words, is the decrease in exhaustivity an artifact of experimental design and data preparation? If so, we should employ a different methodology.
2. Is the underlying assumption of a dependency of posterior beliefs on prior beliefs as given by Bayes' theorem (25) in combination with (27) a reasonable assumption for focus exhaustivity inferences? If not, any attempt to fit a model based on this assumption to the presented data is futile and a vacuos analytical procedure.

In order to tackle these two worries we have conducted an additional experiment. In Experiment 2, we have reduced the number of sliders to two: one for the exhaustive state and one for the sum of all non-exhaustive states, thus forcing possible normalization effects to be independent of domain size. We found that after removing these normalization dependencies we could not observe any significant decrease of exhaustivity, even though prior probabilities on average where still judged to decrease, thus fundamentally contradicting the logic of the RSA model.

Addressing the second concern regarding the general dependency of posteriors on priors, we additionally measured exhaustivity at two different settings for the prior at a fixed domain size. Here, we covered a range of prior probabilities of 30–73%. Considering that at prior values 0 and 100% exhaustivity should eventually reach 100 and 0%, respectively, this range should intuitively suffice to elicit an observable and monotonic variation in the measured response. However, no such variations were found. Moreover, raw slider values were still constant and approximately identical to those of Experiment 1, implying that the methodologies of both experiments most likely show two different facets of one and the same underlying fact, namely that the exhaustivity of focus expressions is approximately constant across different domain sizes and priors.

Our findings therefore largely disagree with the predictions of the RSA model, since the observed drop in exhaustivity computed from normalized slider ratings cannot be attributed to the probabilistic beliefs of the participants. Even more crucial, it seems rather unlikely that one can model our data on narrow focus exhaustivity in a meaningful way with recourse to Bayesian inference in the sense of Equation (25) and Assumption (27) in general, because our data rather suggests that people largely ignore beliefs on prior probabilities of states when interpreting expressions with narrow focus.

These results are surprising given that the RSA model was successful in modeling many—partly unrelated—phenomena. Moreover, the RSA-framework in general is deeply appealing at the conceptual level, so it is really hard to see why the majority of participants should now fail to adhere to the basic Bayes' theorem and ignore valuable information in form of the prior. After all, there is a sense in which the RSA-model is nothing but an implementation of what rational reasoning in a communicative setting is. But if participants interpret focus expression approximately as assumed in the RSA framework, how can the observed independence of exhaustivity from prior probabilities of states be explained?

One explanation that comes to mind is the well-known effect of *base rate neglect* (Kahneman and Tversky, 1973; Pennycook and Thompson, 2017) where people seem to ignore information on the prior probability and prefer judging probability of events based solely on the descriptive information of the actual given case, which in our items would correspond to the question-answer pair. Since people then just disregard prior information, they would essentially always reach at the same exhaustivity on average. However, base rate neglect seems to be cancelable and people can be made aware of the base rate (cf. Birnbaum, 2004), e.g., if people are given multiple tasks with different priors where the prior information is transparently presented. Our experiments are likely to fall in this category, since participants saw three items with different domain sizes and even without fillers, so we made it particularly easy for them to notice the change in domain size and priors. Moreover, in Experiment 2 items for prior elicitation had a very similar structure to their counterparts for posterior elicitation in as they also present descriptive information after the introduction of the base rate. So if base rates in posterior elicitation items are neglected in general, we would have expected participants to neglect base rates in prior elicitation items in a similar way. Since this is not what we observe, base rate neglect alone cannot serve as an explanation of our data.

A similar explanation would be an approach like the one presented in Degen et al. (2015) for their results on scalar implicature. There, participants also gave ratings of posterior probability that showed unexpectedly little variation compared to RSA model predictions. The presented items contained events that were, given their prior probabilities, extremely surprising or “wonky.” The authors reason, roughly, that people in such situations use a uniform back-off prior that replaces the original prior in such situations. Applied to our items in Experiment 2 at *k* = 2, one could assume that in the “none” and “all” condition participants used a back-off prior because the event presented in the respective items is unlikely given those two more extreme priors: Since we use the wordings “usually, all” and “usually, none” to induce a change in priors, participants might always use a uniform prior instead because the descriptive content only mentions one individual. However, like base rate neglect, wonkiness cannot explain the absence of posterior variation, because it is already accounted for in the design of the items for prior elicitation in Experiment 2: There, after reading the introduction of the scenario including the description of the prior by “Usually, some/none/all of his friends [...],” participants where asked to rate prior probabilities given that they already know that one person performed the target action. So, for example, even if the occurrence of the event was extremely unlikely (“Usually, none of his friends Y'ed”), the priors were elicited under the presumption that at least one such event still occurred, thus the prior elicitation items should likewise trigger the use of a back-off prior. As we still observe strong variation in judgements on prior probability the absence of variation in judgements on posterior probability cannot be explained by assuming that participants use a back-off prior.

To summarize, it appears to us that there is no obvious way to account for the fact that both the unequivocally produced variation in prior probability and the variation of domain size did not had any significant effect on exhaustivity. We find this result especially surprising given that the literature on exhaustification of narrow focus not only converges on the verdict that exhaustivity comes about as Gricean quantity implicature, but indeed large parts of the literature use exactly the same derivation for focus exhaustivity as for many phenomena that the RSA model has been successfully applied for. This raises general doubt on modeling exhaustivity implicatures by Bayesian inference in the sense discussed above.

Where to go from here? One way is to reevaluate the RSA program for exhaustivity implicatures. But then, what form of reasoning are exhaustivity implicatures based on? Two possibilities come to mind: 1. Exhaustivity implicatures are a conventionalized implicature which is part of general linguistic knowledge and communication practices. Historically, this conventionalized implicature might have been developed from an originally Bayesian reasoning, an evolutionary process which might not yet be completed for other kinds of implicatures. Since Bayesian reasoning is complex and comparably costly, conventionalization of implicatures might be a logical step in the evolution of language, especially if the respective implicatures are very basic and frequent so that underlying patterns are more likely to be recognized and learned. 2. Given that we already know that people deviate from Bayesian reasoning in some cases (e.g., Kahneman and Tversky, 1973), it might be that people do not employ Bayesian reasoning or conventionalized forms thereof to compute exhaustivity inferences. Still, exhaustivity implicatures are important in communication and thus require a reliable communication channel. One would then have to assume that people use other kinds of reasoning or heuristics in order to solve complex problems like exhaustivity implicatures with less effort^27^.

An alternative way would be to assume that all other exhaustivity inferences do work in the way predicted by RSA models, but that narrow focus exhaustivity inferences are an exception from this general tendency. Of course, besides an explanation of why the latter inferences are special, this would call for a reevaluation of the relation between narrow focus exhaustivity implicatures and other kinds of exhaustivity implicatures.

## Data Availability Statement

The raw data supporting the conclusions of this article will be made available by the authors, without undue reservation.

## Ethics Statement

The studies involving human participants were reviewed and approved by Ethics Committee, University of Graz. The patients/participants provided their written informed consent to participate in this study.

## Author Contributions

AS contributed to the study design, data collection and analysis, and literature review and discussion. EO contributed to the literature review, discussion, and supervision. All authors contributed to the article and approved the submitted version.

## Conflict of Interest

The authors declare that the research was conducted in the absence of any commercial or financial relationships that could be construed as a potential conflict of interest.

## Publisher's Note

All claims expressed in this article are solely those of the authors and do not necessarily represent those of their affiliated organizations, or those of the publisher, the editors and the reviewers. Any product that may be evaluated in this article, or claim that may be made by its manufacturer, is not guaranteed or endorsed by the publisher.

## Footnotes

^1^We do wish to mention that alternative models of deriving quantity implicatures with high level of mathematical sophistication exist in the literature, cf. Benz and van Rooij (2007), Franke (2011), that we will not discuss in this paper.

^2^$P\left(D_{C}\right)$ contains all enumerations of individuals like “Audrey,” “Audrey and Bob,” “Audrey, Bob, and Dale,” etc. formed by groups of individuals of *D*_*C*_.

^3^Whether participants infer meanings by using Bayesian inference, i.e., by using Bayes' theorem *p*(*s*|*e*) ∝ *p*(*e*|*s*) · *p*(*s*), can be checked independently of the RSA framework in empirical terms, cf. e.g., Rohde and Kehler (2014).

^4^Since in all our examples we will use subject focus in non-thetic sentences and the subject will always be a proper name, we will not need to worry about potential mismatches between semantic focus and prosodic stress, cf. Krifka (2008) and Zimmermann and Onea (2011) for an overview discussion.

^5^Although Bergen and Goodman (2015) also model exhaustivity inferences connected with prosodic stress, their work and model crucially rely on listeners uncertainty about speaker knowledge. In the experiments we conduct, we do not elicit any doubt as to whether the speaker is unsure about who participated in a certain activity. Additionally, in another pilot study we experimentally confirmed that stating explicitly the information that the speaker knows exactly who performed the activity does not produce any changes in comparison to the results of the experiments we present in this paper. We therefore conclude that, at least in the phenomena we consider in this work, exhaustivity does not arise by reasoning about the speakers certainty or competency concerning his knowledge about the state of affairs.

^6^Here, *s*_Bob_ is taken to represent the state in which only Bob danced and *s*_Bob&Dale_ is the state in which only Bob and Dale danced. Crucially, these two states are incompatible with each other. By contrast, the meaning of the statement *BOB danced* is compatible with all states in which Bob danced, i.e., *s*_Bob_, *s*_Bob&Dale_, etc.

^7^Design and results of this experiment are shown in Supplementary Material 2.

^8^Since a binomial distribution with *p* = 0.5 is the uniform distribution, this also holds in the case of a uniform distribution of states.

^9^Even if we allow the domain size to be pragmatically limited to a set of a few relevant people known by the speaker, intuitively this number does not seem to be easily determinable nor relevant for the listener.

^10^It should be noted that with inclusion of exclusives Equation (16) cannot be used to compute exhaustivity. We therefore computed complete speaker and listener matrices in order to calculate *E*(*k*).

^11^See also section 4 for a discussion of the applicability of this model to focus exhaustivity in cases of pure prior variation.

^12^After the experiment, participants were asked to indicate their native language and the languages spoken at home when they were children. More than 95% of the participants indicated English only as their native language in this survey. This also holds for Experiment 2.

^13^This friend was chosen randomly from the list of friends present on the occasion.

^14^Some participants, however, might be in doubt on the exact prosody the speaker used. In particular, variation in prosody is conceivable beyond the selection of narrow focus. We thank an anonymous reviewer for highlighting this point. Clearly, using spoken items in similar research may be able to definitively solve this issue. For now, we take prosodic variation to potentially be responsible for some limited noise in our data.

^15^We selected less participants for prior elicitation than for posterior elicitation due to higher redundancy of state information with prior probabilities. In prior elicitation items, the number of possible states is twice as large, so participants rate twice as many states. The increase in information about prior probabilities reduces the number of participants necessary in order to have a sufficiently large statistic.

^16^It is assumed that different names in this experiment do not have any impact on the probability of the states. So for example, the state in which Mary and Peter dance is exactly as probable as the state in which Mary and Frank dance, given no prior information on those individuals, and the same for all other combinations of two individuals in the domain.

^17^Our model is, in a certain way, an intermediate solution between the original RSA model with a fixed α and the one of Franke and Degen (2016). There, in order to capture individual differences, the number of recursions in the model was varied among the population, in contrast to the fixed number of two recursions in the RSA model's listener *L*_2_. The main reason for our choice of model design is that increasing the number of recursions is approximately equivalent to increasing α, e.g., both variations lead to an increase of the probability of the exhaustive state. This avoids computational difficulties arising from implementing further reasoning types with more levels of recursion.

^18^The choice of a different distribution, e.g., like a uniform prior, did not influence the results obtained.

^19^We thank anonymous reviewer for highlighting the possible presence of spillover effects in our experiments.

^20^Additionally, in order to ameliorate spillover effects in terms of accidental low or high slider placement we randomized the order of sliders in every item. This makes it difficult to (partially) remember choices from previous items.

^21^In this paper, all error bars correspond to the standard error of the mean. For reasons of simplicity, for population means, which result from averaging over individual values for participants, we drop the participant index *j*, so e.g., we write *P*_*ik*_ for the population mean 〈_*P*_*ijk*_〉*i*_ and $\underset{i}{\sum }U_{ik}$ for ${\langle \underset{i}{\sum }U_{ijk}\rangle }_{i}$.

^22^An ANOVA revealed that there is no dependence on the type of scenario, so we collapsed over the three scenarios.

^23^For a visualization of the corresponding changes in item design, compare Supplementary Figures 3, 5 for prior elicitation of Experiments 1 and 2.

^24^For the “none” condition we recruited more participants than for the “all” condition because the resulting posterior probability for the “none” condition is expected to be much closer to the original “some” condition. In order to resolve possible differences between them equally good, we increased the statistics.

^25^Condition (27) is a relatively weak assumption. To see why, consider that the utterance *foc*_*A*_ is always logically compatible with both *s*_*A*_ and *s*_*AB*_. So if the speaker uses *foc*_*A*_ to communicate the actual state *s*_*AB*_, there is always a certain risk that the listener believes *s*_*A*_ instead. If the listener takes prior probabilities into account in a rational way, this risk should increase with the prior probability *p*(*s*_*A*_). If, in turn, speakers act rational, they should therefore be less likely to use *foc*_*A*_ in state *p*(*s*_*AB*_) when *p*(*s*_*A*_) increases.

^26^For these approximate predictions we used κ = 1, which was approximately fulfilled in Experiment 1.

^27^See e.g., Tversky and Kahneman (1974) for examples on general strategies and heuristics people employ when confronted with complex problems with a probabilistic nature.
