# Supplementary material for: Are Narrow Focus Exhaustivity Inferences Bayesian Inferences?
Source: Front Psychol. 2021 Aug 4;12:677223. doi: 10.3389/fpsyg.2021.677223 (PMC8371404; doi:10.3389/fpsyg.2021.677223)
Supplement: Supplementary file 1 [file Presentation_1.pdf]

Appendices

APPENDIX 1. DATA ANALYSIS DETAILS

Table S1: Means of marginal posteriors and 95% HDIs of parameters for prior probabilities of Experiment 1.

	HDI min	mean	HDI max
σ	0.417	0.441	0.466
$\vec{\mu_2}$	[0.0902,0.481,0.283]	[0.121,0.542,0.337]	[0.155,0.597,0.393]
$\vec{\mu_3}$	[0.0517,0.319,0.326,0.136]	[0.0731,0.372,0.379,0.176]	[0.0963,0.426,0.433,0.217]
$\vec{\mu_4}$	[0.0383,0.181,0.314,0.182,0.0956]	[0.0553,0.225,0.365,0.226,0.128]	[0.0737,0.268,0.416,0.269,0.163]
w	8.3	10	11.8
κ	1.01	1.02	1.03

Table S2: Means of marginal posteriors and 95% HDIs of parameters for posterior probabilities of Experiment 1.

	HDI min	mean	HDI max
σ	0.485	0.519	0.553
μ_α	2.09	2.81	3.54
σ_α	2.34	3.31	4.34
κ	1.04	1.07	1.1

APPENDIX 2. DETERMINATION OF THE LEXICON

Participants, materials and procedures

In order to determine the lexicon $\mathcal{L}$ of the RSA model, we conducted a production experiment. We recruited 59 particiants through Prolific Academic of which four were excluded because of having provided an answer that either did not address the QUD directly or that was false in the context of the QUD. We collected responses for 36 different items in return for a small payment. We used three different domain sizes $k \in \{2, 4, 8\}$ and varied the number of individuals in the target set in the following way:

(1) for $k = 2$: $n_{targets} \in \{0, 1, 2\}$
for $k = 4$: $n_{targets} \in \{0, 1, 2, 4\}$
for $k = 8$: $n_{targets} \in \{0, 1, 3, 6, 7\}$

Tab. S3 gives an overview of the elements which were combined to items.

Table S3: Possible combination of elements used in the items for Experiment 3.

Lexicalisations	Domain sizes	Target set sizes
Being on vacation<br>Halloween dinner party<br>Movie night	$k \in \{2, 4, 8\}$	see (1)

Like in the posterior elicitation items of Experiment 1, every participant saw three items, consisting of three different scenarios in random order, each one for a different domain size, but with possibly equal target set sizes.
In contrast to the posterior elicitation items of Experiment 1, after the question, an empty input field was added in which participants were asked to introduce a response they would expect in the conversation. The true state, i.e., the number of friends engaged in the action, was indicated to the participants by coloring the names of friends mentioned by the protagonist with green (part of the target set) and blue (not part of the target set). Fig. S1 shows one of the items.

Kevin loves movie nights with his friends, where everybody brings a movie he likes to watch. Usually, some of his friends bring a horror movie. Imagine you overhear the following conversation between him and his colleague Jeff:

Kevin: *Yesterday, I invited some friends over for movie night. David, James, Michael, and William came.*
Jeff: *Which of them brought a horror movie?*
Kevin: ______________________

In fact, the persons marked green (if any) brought a horror movie while the persons marked blue (if any) brought a comedy movie. Given that Kevin is known to be truthful, insert an answer you would expect Kevin to give to Jeff's question in the text above.

Figure S1: One of the three scenarios of the experiment with domain size $k = 4$

Results

Fig. S2 shows the frequencies of different expression types for $n_{targets} > 0$ for the three different domain sizes separately.

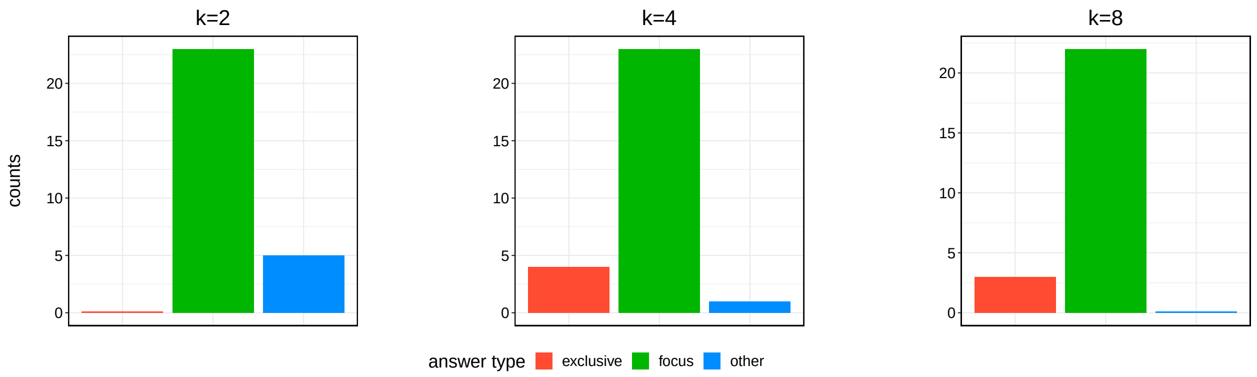

Figure S2: Frequencies of the different expression types used as answers by the participants.

We grouped the answers in the following way:

(2) *Exclusive* := {'Only Peter went camping', 'Only Peter', ...}
Focus := {'Peter went camping', 'Peter', ...}
Other := {'Everyone except/but', 'Both of them', ...}

The vast majority of answers are narrow focus expressions. Exclusives are very rare and are only used at domain sizes $k > 2$. All remaining answer types have a very low frequency, which is why they were grouped together.

APPENDIX 3. EXAMPLES OF ITEMS USED IN EXPERIMENTS 1 AND 2

Experiment 1

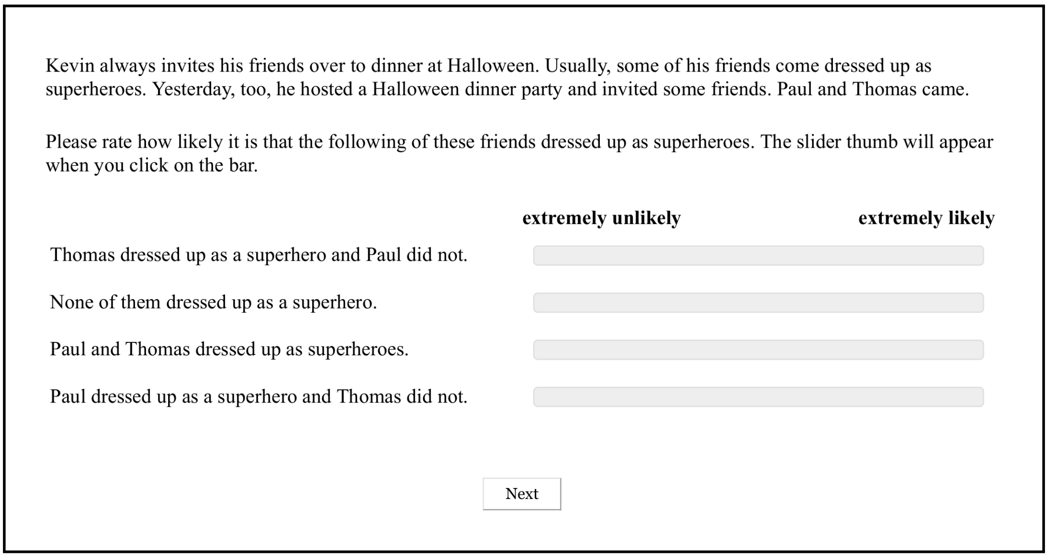

Figure S3: One of the three scenarios of Experiment 1 with domain size $k = 2$ used to elicit prior probabilities.

Experiment 2

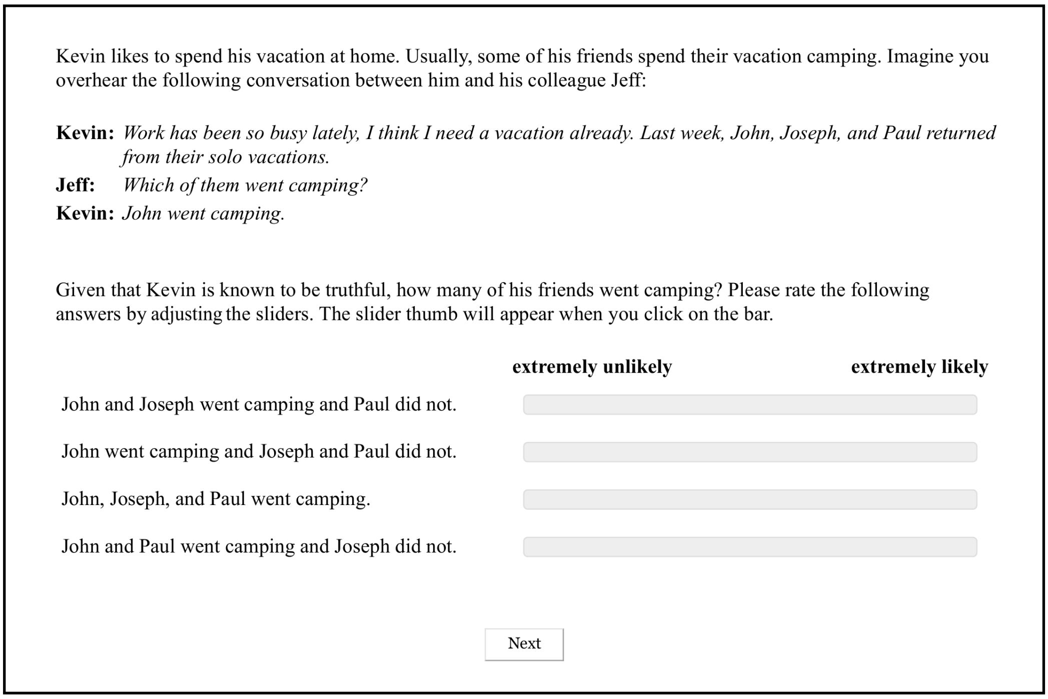

Figure S4: One of the three scenarios of Experiment 1 with domain size $k = 3$ used to elicit posterior probabilities.

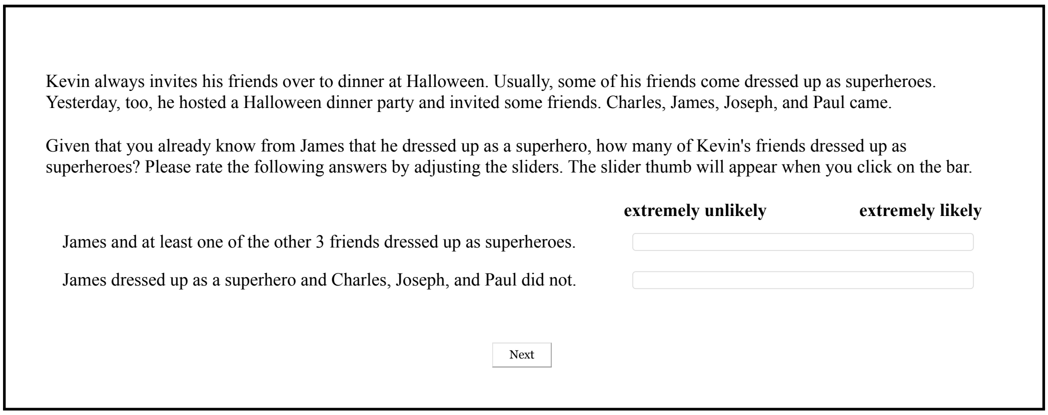

Figure S5: One of the three scenarios of Experiment 2 with domain size $k = 4$ used to elicit prior probabilities.

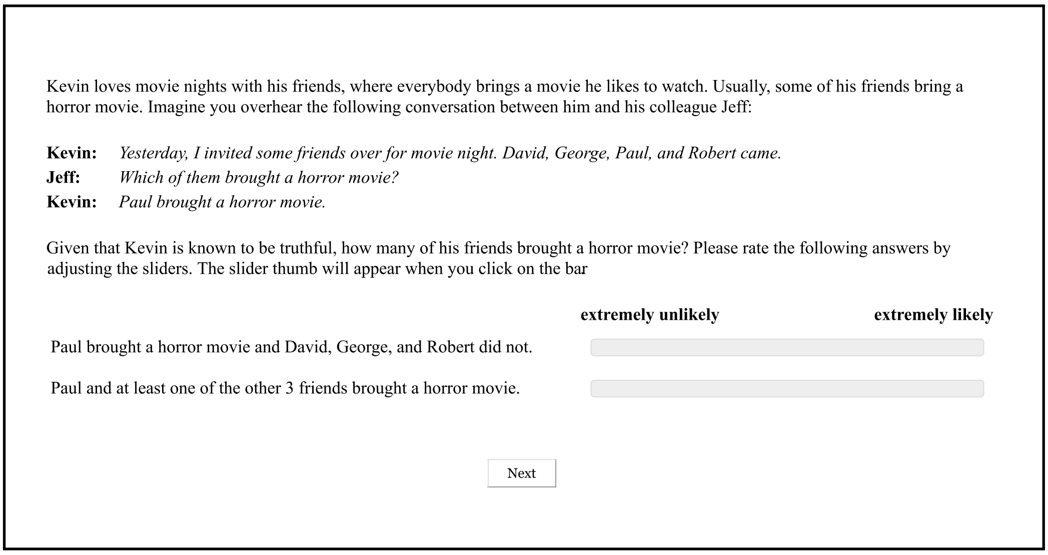

Figure S6: One of the three scenarios of Experiment 2 with domain size $k = 4$ used to elicit posterior probabilities.
